# Acute effects of strength exercise with blood flow restriction on vascular function of young healthy males

**DOI:** 10.1590/1677-5449.011017

**Published:** 2018

**Authors:** Francesco Pinto Boeno, Thiago Rozales Ramis, Juliano Boufleur Farinha, Leandro Silva de Lemos, Niara da Silva Medeiros, Jerri Luiz Ribeiro

**Affiliations:** 1 Universidade Federal do Rio Grande do Sul – UFRGS, Programa de Pós-graduação em Ciências do Movimento Humano, Porto Alegre, RS, Brasil.; 2 Universidade Regional Integrada do Alto Uruguai e das Missões – URI, Departamento de Ciências da Saúde, São Luiz Gonzaga, RS, Brasil.; 3 Centro Universitario Metodista – IPA, Porto Alegre, RS, Brasil.

**Keywords:** strength training, nitric oxide, oxidative stress, blood flow restriction

## Abstract

**Background:**

Strength training with blood flow restriction (STBFR) provokes similar neuromuscular adaptations to traditional strength training using low training loads. However, there is a need for better understanding of the repercussions for antioxidant parameters and vascular function.

**Objectives:**

The objective of the present study was to investigate the effects of a session of low intensity strength training with blood flow restriction, compared with high intensity and low intensity strength training without blood flow restriction, on the levels of nitric oxide products and antioxidant enzyme activity in healthy young men.

**Methods:**

Eleven young men performed three strength exercise sessions: low intensity with blood flow restriction (LIBFR), high intensity (HI), and low intensity (LI). Activity of the antioxidant enzymes catalase (CAT) and superoxide dismutase (SOD) was assessed and metabolites of nitric oxide (NOx) were assayed before and after each session.

**Results:**

There were no changes to NOx plasma levels under the different exercise conditions (p > 0.05). However, SOD activity exhibited a significant reduction after the LIBFR condition (p < 0.05), while CAT activity reduced significantly after the LI condition (p < 0.05).

**Conclusions:**

The results of this study suggest that one session of low intensity strength training with blood flow restriction does not reduce bioavailability of nitric oxide or induce redox imbalance in healthy young men.

## INTRODUCTION

 Strength training with blood flow restriction (STBFR) can provoke significant strength and muscle hypertrophy gains using low training loads in combination with an occlusive component during exercise. 1 Several studies have suggested that musculoskeletal adaptations in response to STBFR occur at intensities at 20-50% of one repetition maximum (1RM) and at a similar proportion to those observed in traditional strength training, in which loads of around 80% of 1RM are generally used. 1 ^-^ 3 It has therefore been proposed that different molecular mechanisms are involved in the adaptations provoked by STBFR. There appears to be recruitment of motor units that are only activated at elevated work intensities, due to the low oxygen availability; 1 positive signaling in the mTOR activation cascade; 4 myostatin inhibition; 3 and increased release of growth hormone. 5

 Therefore, STBFR could prove to be an important tool for health promotion in individuals with restrictions preventing high intensity exercise. However, few studies have investigated the repercussions of STBFR on prooxidant and antioxidant agents or the relationship with vascular function. 2 ^,^ 6 Acute compromise of vascular function has been shown to be an important prognostic factor of the incidence of cardiovascular events. 7 Reduced bioavailability of nitric oxide may trigger a hypertensive and prooxidant response, exponentially increasing cardiovascular risk in both healthy and diseased individuals. 8

 Increased production of reactive oxygen species affects vascular reactivity, accelerating the atherosclerotic process by creating an imbalance between prooxidant and antioxidant systems, leading to higher cardiovascular risk. 9 ^,^ 10 It is therefore important to understand the behavior of variables related to vascular function in reaction to an STBFR session, since this is an alternative to traditional strength training with substantially lower intensity and volume. The objective of this study is to investigate the effects of a low intensity strength training exercise session with blood flow restriction, comparing it to high and low intensity strength exercise without blood flow restriction in terms of nitric oxide byproduct levels and antioxidant enzyme activity in healthy young men. 

## METHODS

 Eleven young men were recruited via printed media and social networks. They were physically active, had no history of diseases, and took part in the study voluntarily. The study protocol was approved by the Ethics Committee at the Centro Universitário Metodista (IPA), under registration number 361/2012. 

### Experimental design

 The design is a randomized crossover study, comprising 5 days of assessments, separated by 72-hour intervals between each. Volunteers undertook an experimental protocol consisting of 2 days of assessments with three different conditions of strength exercise: low intensity with blood flow restriction (LIBFR), high intensity (HI), and low intensity (LI). 

 At the first visit, anthropometric assessments were conducted and participants were familiarized with the one maximum repetition (1RM) test for leg press and bilateral elbow flexion exercises. At the second visit, the 1RM test was conducted for both exercises. After these initial assessments, the experimental protocols were scheduled. All protocols were conducted during the morning and consisted of performing strength exercises, with or without blood flow restriction. Before and immediately after interventions, blood samples were drawn by a trained professional for determination of blood variables. 

### Anthropometric assessment

 Body composition was determined using the five-component method, according to standards set by the International Society for the Advancement of Kinanthropometry, as described elsewhere. 11

### 1RM test

 After familiarization, the test of maximum dynamic strength was conducted using free weights for elbow flexions and by loading the leg press machine. After a warm-up period, intensity was set at approximately 80% of predicted 1RM. If the volunteer was able to perform more than one repetition, then the load was increased by around 5% until he was no longer able to complete the movement with good technique. A maximum of five attempts were made, with a rest interval of at least 3 minutes between attempts. 12

### Exercise protocols

 When they arrived at the laboratory in the morning, participants were positioned in decubitus dorsal and remained at absolute rest for 10 minutes for measurement of arterial blood pressure at rest. This was conducted using the auscultatory method with a mercury column sphygmomanometer. Arterial blood pressure was measured to enable the magnitude of blood flow restriction to be calculated. Next, after randomization by lots, the volunteers performed exercises in one of the conditions described above. Interventions consisted of elbow flexion and leg press, both performed bilaterally. The exercise volume for each of the three conditions was set at four series of maximum repetitions, with 1 minute intervals between series. The speed of movements was controlled by a metronome at a cadence of 1 second for each phase of the movement. Exercise intensity was set as 30% of 1RM for LIBFR and LI, and 80% of 1RM for HI. 

 For LIBFR, inflatable cuffs were fitted to the proximal part of the volunteers’ upper limbs, and pressure was set at 20 mmHg below systolic arterial blood pressure (SBP) for elbow flexion exercises. After the exercise, the cuffs were removed and fitted to the proximal portion of the lower limbs, and inflated to a pressure 20 mmHg greater than SBP. 2 Blood flow restriction was maintained during the intervals between each series. After each maximum series, a finger oximeter (New Tech, model PM100C, Brazil) was used to guarantee partial blood flow restriction. If the oximeter signal was absent or oxygenation was below 90%, the pressure in the cuff was reduced by 5 mmHg until the signal was detected again, to ensure that blood flow was not halted completely. 2 ^,^ 13 For HI and LI exercises, cuffs were merely fitted to the volunteers in the same places. 

### Blood sampling and biochemical analyses

 Blood samples were taken from the antecubital area before and soon after each exercise condition. Samples were drawn into tubes containing EDTA, centrifuged and stored at -80 °C for later analysis. 

 Plasma concentrations of nitrites and nitrates (NOx) were determined as described previously, 14 and the results expressed in μM/mL. Superoxide dismutase (SOD) enzyme activity was measured by adrenochrome formation. 15 SOD levels are expressed as SOD/mg of protein. Plasma activity of the enzyme catalase (CAT) was assayed using the method described by Aebi 16 and results were expressed in U/mg of protein. 

### Statistical analysis

 Data distributions were analyzed using the Shapiro-Wilk test. Mixed-model analysis of variance (ANOVA) was used for intergroup and intragroup comparisons. The cutoff for significance adopted was p < 0.05 and the Bonferroni post hoc test was used when necessary. All data were analyzed using the Statistical Package for Social Sciences, version 20. Data are expressed as means and standard deviations (SD). 

## RESULTS

Table 1 lists the variables used to characterize the sample. 

**Table 1:** Characteristics of the sample.

**Variable**	**Mean ± SD**
Age (years)	23.72±3.49
Body mass (kg)	81.55±6.10
Fat mass (%)	25.91±3.93
Fat free mass (%)	46.59±2.90

SD = standard deviation.

 For both exercises, the number of repetitions performed in each series was higher in the LI condition than in the other conditions (p < 0.05). The number of repetitions of the elbow flexion exercise performed in the LIBFR condition was significantly higher than the number performed in the HI condition, whereas for the leg press exercise, the number of repetitions performed did not differ between the conditions. Mean 1RM values and mean number of repetitions performed in the series are shown in Table 2 . 

**Table 2:** 1RM values and number of repetitions for each protocol.

	**LIBFR**	**HI**	**LI**
1RM			
Elbow flexion	18±3.19	18±3.19	18±3.19
Leg press	172.52±29.20	172.52±29.20	172.52±29.20
Repetitions			
Elbow flexion	21.2±5.3 ^#^	5.7±3	28.1±12.5 ^#^
Leg press	20.3±6.3	22±10.8	89.4±23.3 ^*^

# p < 0.05 in relation to condition HI;

* p < 0.05 in relation to other conditions; LIBFR: low intensity with blood flow restriction; HI: high intensity; LI: low intensity; 1RM: one repetition maximum.

**Figure 1:**
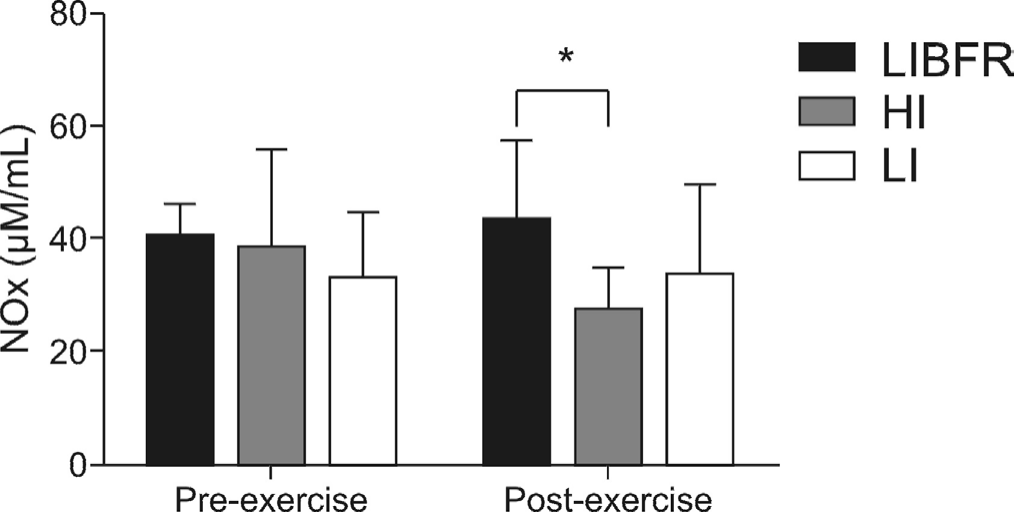
Plasma levels of metabolites of nitric oxide (NOx) before and after exercise. *Intragroup difference (p < 0.05).

 No significant differences were detected in baseline NOx levels between the three conditions: 38.8±18 µM/mL in condition HI; 40.6±6 µM/mL in condition LIBFR; and 33.2±13.7µM/mL in condition LI. There was no modulation in these levels from pre-exercise to post-exercise in any of the three experimental conditions (p > 0.05). However, post-exercise plasma NOx concentrations were significantly reduced in condition HI, when compared with condition LIBFR (p < 0.05). Condition LI did not exhibit modulations between pre-exercise and post-exercise or in comparison with the other conditions. The changes in plasma NOx levels are illustrated in Figure 1 . 

**Figure 2:**
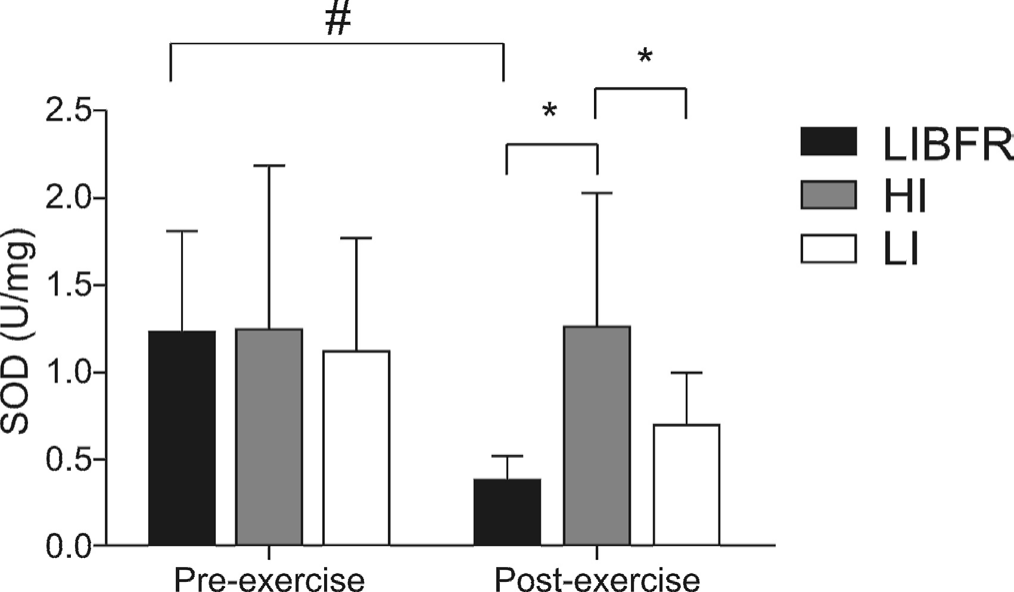
Superoxide dismutase (SOD) activity before and after exercise. *Intergroup difference (p < 0.05); # Intragroup difference (p < 0.05).

 There were no significant differences between the three conditions in SOD activity at rest. However, in the LIBFR condition, SOD activity was reduced after exercise (p < 0.05). In condition HI, SOD activity was significantly higher than in the other conditions soon after the exercise session ( Figure 2 ). 

**Figure 3:**
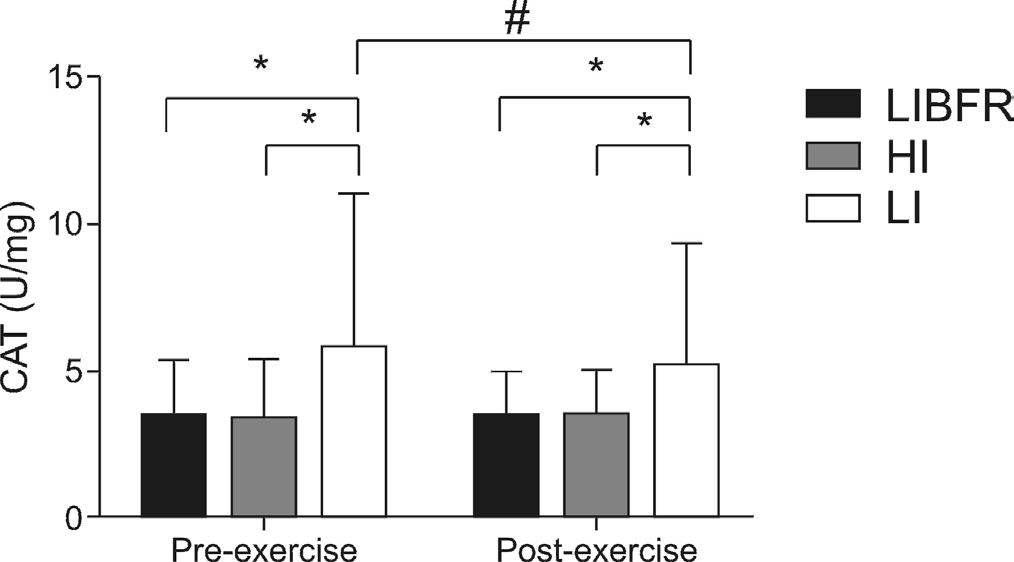
Plasma levels de catalase (CAT) before and after exercise. *Intergroup difference (p < 0.05); # Intragroup difference (p < 0.05).

 Activity of the CAT antioxidant enzyme was significantly higher in condition LI than in the others at rest and after exercise. There was a significant reduction in CAT activity in condition LI soon after the end of the session. There were no changes in CAT activity in conditions LIBFR or HI at any of the sample times ( Figure 3 ). 

## DISCUSSION

 The objective of this study was to investigate the effect on nitric oxide byproduct levels and antioxidant enzyme activity of a low intensity strength training exercise session with blood flow restriction (LIBFR), compared with high intensity (HI) and low intensity (LI) strength exercises without blood flow restriction, in healthy young men. The main findings show that: 1) one session of LIBFR strength exercise is not capable of modulating plasma NOx levels; 2) LIBFR strength exercise significantly reduced SOD activity and; 3) neither HI nor LIBFR exercises modulate CAT activity. 

 NOx are the final products of metabolism of nitric oxide, which plays a double physiological role since, while it has a function as a vasodilator, antihypertensive, and antiatherosclerotic, it can also cause oxidative damage through formation of peroxynitrite radicals in the presence of the superoxide anion. 8 ^,^ 17 As stated earlier, plasma levels of NOx did not change in response to LIBFR. However, when compared with the levels in condition HI, these levels were significantly higher after exercise. It has already been demonstrated that traditional high intensity strength exercise can acutely compromise flow-mediated vasodilation, reducing NOx levels in sedentary people. 18 ^,^ 19 In such a scenario, reduced nitric oxide bioavailability, combined with the high intensity, could be the result of two factors: increased sympathetic activation during exercise and consequent acetylcholine-mediated vasoconstriction 20 and increased muscle compression of blood vessels when large muscle groups are recruited. 19 ^,^ 20

 In the present study, a significant reduction in SOD activity was observed in the LIBFR condition. Since NOx levels were not reduced after the LIBFR protocol, it could suggest that this session was not capable of causing significant production of reactive oxygen species and a consequent transitory degree of oxidative stress, which therefore did not demand an increase in SOD activity to eliminate a possible excess of superoxide. 8 ^,^ 10 Along the same lines, a study that used a very similar LIBFR protocol did not detect any increase in blood levels of pro-oxidative or antioxidant markers soon after an exercise session, confirming the fact that this type of session is not capable of provoking a significant degree of oxidative stress. 2

 In this study, CAT activity did not exhibit changes in the LIBFR and HI protocols. The CAT enzyme reduces hydrogen peroxide (H_2_O_2_) levels to water (H_2_ O) and oxygen (O_2_). 10 The low volume of exercise and the magnitude of muscle injury may explain these results. Child et al. conducted a study in which participants performed 70 maximum eccentric knee extension contractions, observing significant increase in muscle damage, represented by creatine kinase (CK) and, consequently, increased antioxidant protection. 21 Therefore, our findings may also be related to the low impact of the exercise protocols on muscle damage induced by the exercise. 

 Certain limitations are relevant in the context of this study. For example, vascular function was only measured using biochemical variables, while image analysis or plethysmography could provide a foundation for more striking inferences from our results. Additionally, measurement of plasma CK levels or other markers of muscle damage could indicate the metabolic magnitude of the different conditions. 

## FINAL COMMENTS

 On the basis of the results of this study it can be inferred that one low intensity strength training session with blood flow restriction does not reduce nitric oxide bioavailability or induce redox imbalance in healthy young men. As perspectives, future studies should assess vascular function in response to strength training with blood flow restriction in greater depth. 
