# Supplementary material for: Acute effects of strength exercise with blood flow restriction on vascular function of young healthy males
Source: J Vasc Bras. 2018 Apr-Jun;17(2):122–7. doi: 10.1590/1677-5449.011017 (PMC6205709; doi:10.1590/1677-5449.011017)
Supplement: Translation

# Translation: Efeito agudo do exercício de força com restrição do fluxo sanguíneo sobre parâmetros antioxidantes em indivíduos jovens saudáveis

**Authors:** Francesco Pinto Boeno, Thiago Rozales Ramis, Juliano Boufleur Farinha, Leandro Silva de Lemos, Niara da Silva Medeiros, Jerri Luiz Ribeiro

**Affiliations:** 1 Universidade Federal do Rio Grande do Sul – UFRGS, Programa de Pós-graduação em Ciências do Movimento Humano, Porto Alegre, RS, Brasil.; 2 Universidade Regional Integrada do Alto Uruguai e das Missões – URI, Departamento de Ciências da Saúde, São Luiz Gonzaga, RS, Brasil.; 3 Centro Universitario Metodista – IPA, Porto Alegre, RS, Brasil.

**Keywords:** treinamento de força, óxido nítrico, stress oxidativo, restrição do fluxo sanguíneo

## Abstract

**Contexto:**

O treinamento de força com restrição do fluxo sanguíneo (TFRFS) promove adaptações neuromusculares semelhantes às do treinamento de força tradicional utilizando pequenas cargas de treinamento. No entanto, sua repercussão sobre parâmetros antioxidantes e sobre a função vascular precisa ser mais bem compreendida.

**Objetivos:**

O objetivo do presente estudo foi investigar o efeito de uma sessão de exercício de força de baixa intensidade com restrição do fluxo sanguíneo, em comparação ao exercício de força de alta intensidade e de baixa intensidade sem restrição do fluxo sanguíneo, sobre os níveis de subprodutos do oxido nítrico e a atividade de enzimas antioxidantes em jovens saudáveis.

**Métodos:**

Onze indivíduos jovens realizaram três sessões de exercício de força: baixa intensidade com restrição do fluxo sanguíneo (BIRFS), alta intensidade (AI) ou baixa intensidade (BI). Foram avaliadas a atividade das enzimas antioxidantes catalase (CAT), superóxido dismutase (SOD) e dos metabólitos do óxido nítrico (NOx).

**Resultados:**

Não houve modificações nos níveis plasmáticos de NOx nas diferentes condições de exercício (p > 0,05). A atividade da SOD apresentou uma diminuição significativa na condição BIRFS (p < 0,05). A atividade da CAT diminuiu significativamente na condição BI (p < 0.05).

**Conclusões:**

A partir do presente estudo sugere-se que uma sessão de treinamento de força de baixa intensidade com restrição do fluxo sanguíneo não reduz a biodisponibilidade do óxido nítrico, bem como não induz desequilíbrio redox em indivíduos jovens saudáveis.

## INTRODUÇÃO

 O treinamento de força com restrição do fluxo sanguíneo (TFRFS) tem promovido ganhos significativos de força e hipertrofia muscular através do uso de baixas cargas de treinamento associadas a um componente oclusivo durante a realização do exercício 1 . Diferentes estudos sugerem que as adaptações musculoesqueléticas ao TFRFS ocorrem em intensidades entre 20-50% de uma repetição máxima (1RM) e em proporção semelhante às observadas no treinamento de força tradicional, no qual usualmente são utilizadas cargas em torno de 80% de 1RM 1 ^-^ 3 . Nesse sentido, propõe-se que diferentes mecanismos moleculares estão envolvidos nas adaptações ao TFRFS. Parece haver o recrutamento de unidades motoras ativadas somente em elevadas intensidades de trabalho, devido à baixa disponibilidade de oxigênio 1 ; sinalização positiva na cascata de ativação da mTOR 4 ; inibição de miostatina 3 ; além da elevação na secreção de hormônio do crescimento 5 . 

 Dessa forma, o TFRF pode ser uma importante ferramenta na promoção de saúde em indivíduos com alguma restrição a elevadas intensidades do exercício. No entanto, poucos estudos têm investigado a repercussão do TFRFS sobre agentes pró-oxidantes e antioxidantes, bem como sua relação com a função vascular 2 ^,^ 6 . Comprometimentos agudos na função vascular têm se mostrado um importante fator prognóstico na incidência de eventos cardiovasculares 7 . A redução da biodisponibilidade do óxido nítrico pode deflagrar uma resposta hipertensiva e pró-oxidante, aumentando exponencialmente o risco cardiovascular tanto em indivíduos saudáveis quanto doentes 8 . 

 O aumento na produção de espécies reativas de oxigênio repercute na reatividade vascular, acelerando o processo aterosclerótico através do desbalanço entre os sistemas pró e antioxidante, acarretando um elevado risco cardiovascular 9 ^,^ 10 . Portanto, é importante compreender o comportamento de variáveis associadas à função vascular frente a uma sessão de TFRFS, pois este oferece uma alternativa ao treinamento de força tradicional, com intensidade e volume substancialmente menores. O presente estudo tem como objetivo investigar o efeito de uma sessão de exercício de força de baixa intensidade com restrição do fluxo sanguíneo, em comparação ao exercício de força de alta e de baixa intensidade sem restrição do fluxo sanguíneo, sobre os níveis de subprodutos do óxido nítrico e a atividade de enzimas antioxidantes em jovens saudáveis. 

## MÉTODOS

 Onze indivíduos jovens do sexo masculino, recrutados através de mídia impressa e redes sociais, fisicamente ativos e sem histórico de doenças, voluntariaram-se a participar do presente estudo. O presente trabalho foi aprovado pelo Comitê de Ética do Centro Universitário Metodista (IPA), sob o registro 361/2012. 

### Desenho experimental

 Este estudo caracteriza-se como randomizado cruzado, composto por 5 dias de avaliações separados por 72 horas de intervalo entre si. Os voluntários foram submetidos a um protocolo experimental que consistiu em 2 dias de avaliações e três condições de exercício de força: baixa intensidade com restrição de fluxo sanguíneo (BIRFS), alta intensidade (AI) e baixa intensidade (BI). 

 Na primeira avaliação, foram realizadas a avaliação antropométrica e uma familiarização ao teste de uma repetição máxima (1RM) para os exercícios *leg press* e flexão bilateral de cotovelos. Na segunda visita, foi executado o teste de 1RM para os respectivos exercícios. Após a realização dessas primeiras avaliações, foram agendados os protocolos experimentais. A aplicação dos protocolos ocorreu necessariamente no turno da manhã e consistiu na realização do exercício de força, com ou sem restrição do fluxo sanguíneo. Antes e imediatamente após as intervenções, uma coleta de sangue foi realizada por um profissional habilitado para determinação das variáveis sanguíneas. 

### Avaliação antropométrica

 A composição corporal foi determinada através do método de cinco componentes, seguindo-se as normas da Sociedade Internacional para o Avanço da Cineantropometria, como previamente descrito 11 . 

### Teste de 1RM

 Após familiarizações, o teste de força dinâmica máxima foi realizado com uso de pesos livres para o exercício de flexão de cotovelos e de carga para o equipamento *leg press*. Após um período de aquecimento, a intensidade foi fixada em aproximadamente 80% de 1RM prevista. Caso o voluntário conseguisse realizar mais de uma repetição, a carga era aumentada em cerca de 5% até que o sujeito não conseguisse realizar o movimento completo com boa técnica. Foram realizadas no máximo cinco tentativas, sendo respeitados pelo menos 3 minutos de descanso entre as tentativas 12 . 

### Protocolos de exercício

 Ao chegarem ao laboratório, necessariamente no turno da manhã, os participantes foram posicionados em decúbito dorsal, permanecendo em repouso absoluto por 10 minutos para que a pressão arterial de repouso fosse mensurada. Para tal, foi utilizado o método auscultatório através de um esfigmomanômetro com coluna de mercúrio. A pressão arterial foi medida para determinação da magnitude da restrição do fluxo sanguíneo. Em seguida, através de randomização por sorteio, os voluntários realizaram uma das condições de exercício supracitadas. As intervenções foram compostas pelos exercícios de flexão do cotovelo e *leg press*, ambos realizados de forma bilateral. O volume determinado nas três condições foi de quatro séries de repetições máximas para cada exercício, com intervalo de 1 minuto entre as séries. A execução do movimento foi controlada por um metrônomo com cadência de um segundo para cada fase do movimento. A intensidade do exercício foi estabelecida em 30% de 1RM nas condições BIRFS e BI, e de 80% de 1RM na condição AI. 

 Na condição BIRFS, foram fixados manguitos infláveis na porção proximal dos membros superiores dos indivíduos, sendo determinada uma pressão de 20 mmHg abaixo da pressão arterial sistólica (PAS) para execução do exercício de flexão do cotovelo. Ao final do exercício, os manguitos eram retirados e fixados na porção proximal dos membros inferiores, sendo inflados a uma pressão de 20 mmHg acima da PAS 2 . A restrição do fluxo sanguíneo era mantida durante o intervalo de cada série. Para garantir a restrição parcial do fluxo sanguíneo, foi utilizado um oxímetro de dedo (New Tech modelo PM100C, Brasil) após cada série máxima. Em caso de ausência de sinal no equipamento ou oxigenação abaixo de 90%, a pressão no manguito era reduzida em 5 mmHg até a detecção de sinal, dessa forma assegurando-se que o fluxo sanguíneo não fosse completamente interrompido 2 ^,^ 13 . Na condição AI e BI, os manguitos foram apenas fixados nos voluntários na mesma região. 

### Coleta sanguínea e análises bioquímicas

 Amostras de sangue foram coletadas da região antecubital antes e logo após cada condição de exercício. As amostras foram armazenadas em tubos contendo EDTA, centrifugadas e armazenadas a -80 °C para posterior análise. 

 As concentrações plasmáticas de nitritos e nitratos (NOx) foram determinadas conforme previamente descrito 14 , e os resultados expressos em μM/mL. A atividade da enzima superóxido dismutase (SOD) foi mensurada através da formação de adrenocromo 15 . Os valores de SOD estão expressos em SOD/mg de proteína. A atividade plasmática da enzima catalase (CAT) foi mensurada conforme o método descrito por Aebi 16 , estando os resultados expressos em U/mg de proteína. 

### Análise estatística

 A distribuição dos dados foi avaliada através do teste de Shapiro-Wilk. Foi utilizada a análise de variância (ANOVA) de modelo misto para comparações entre e intra-grupos. Foi adotado um p < 0,05 como significativo e utilizado o teste *post hoc* de Bonferroni quando necessário. Todos os dados foram analisados pelo programa estatístico Statistical Package for Social Sciences 20. Os dados estão apresentados como média e desvio padrão (DP). 

## RESULTADOS

 A Tabela 1 apresenta as variáveis de caracterização da amostra. 

**Tabela 1:** Caracterização da amostra.

**Variável**	**Média ± DP**
Idade (anos)	23,72±3,49
Massa corporal (kg)	81,55±6,10
Massa adiposa (%)	25,91±3,93
Massa livre de gordura (%)	46,59±2,90

DP = desvio padrão.

 Em ambos os exercícios, o número de repetições realizadas a cada série foi maior na condição BI quando comparado às das demais condições (p < 0,05). Na condição BIRFS, o número de repetições concluídas foi significativamente maior para o exercício de flexão do cotovelo em comparação à condição AI, enquanto que, na execução do exercício de *leg press* , o número de repetições executadas não diferiu entre as condições. A média dos valores de 1RM e a média de repetições realizadas ao longo das séries estão demonstradas na Tabela 2 . 

**Tabela 2:** Valores de 1RM e número de repetições realizadas em cada protocolo.

	**BIRFS**	**AI**	**BI**
**1RM**			
Flexão de cotovelo	18±3,19	18±3,19	18±3,19
*Leg press*	172,52±29,20	172,52±29,20	172,52±29,20
**Repetições**			
Flexão de cotovelo	21,2±5,3 ^#^	5,7±3	28,1±12,5 ^#^
*Leg press*	20,3±6,3	22±10,8	89,4±23,3 ^*^

# p < 0,05 em relação à condição AI;

* p < 0,05 em relação às demais condições; BIRFS: baixa intensidade com restrição do fluxo sanguíneo; AI: alta intensidade; BI: baixa intensidade; 1RM: uma repetição máxima.

 Não foram encontradas diferenças significativas nos níveis basais de NOx entre as três condições: 38,8±18 µM/mL na condição AI; 40,6±6 µM/mL na condição BIRFS; 33,2±13,7µM/mL na condição BI. Não houve modulação desses níveis entre os momentos pré e pós-exercício nas três condições experimentais (p > 0,05). Entretanto, as concentrações plasmáticas de NOx no momento pós-exercício mostraram-se significativamente reduzidas na condição AI, quando comparadas às da condição BIRFS (p < 0,05). A condição BI não apresentou modulações entre os momentos ou diferença quando comparada à das demais condições. As alterações nos níveis plasmáticos de NOx estão demonstradas na Figura 1 . 

**Figura 1:** Níveis plasmáticos de metabólitos do óxido nítrico (NOx) pré e pós-exercício. *Diferença intergrupo (p < 0,05).

 A atividade da SOD não apresentou diferença significativa entre as três condições durante o repouso. Contudo, foi encontrada uma diminuição dessa atividade na condição BIRFS no momento pós-exercício (p < 0,05). Na condição AI, a atividade da SOD se mostrou significativamente aumentada em relação às demais condições logo após a sessão de exercício ( Figura 2 ). 

**Figura 2:** Atividade da superóxido dismutase (SOD) pré e pós-exercício. *Diferença intergrupo (p < 0,05); # Diferença intragrupo (p < 0,05).

 A atividade da enzima antioxidante CAT mostrou-se significativamente maior na condição BI em relação às demais durante o repouso e após o exercício. Houve uma diminuição significativa da atividade da CAT na condição BI logo ao fim da sessão. Nas condições BIRFS e AI, a atividade da CAT não sofreu alteração em nenhum dos momentos ( Figura 3 ). 

**Figura 3:** Níveis plasmáticos de catalase (CAT) pré e pós-exercício. *Diferença intergrupo (p < 0,05); # Diferença intragrupo (p < 0,05).

## DISCUSSÃO

 O presente estudo objetivou investigar o efeito de uma sessão de exercício de força de baixa intensidade com restrição do fluxo sanguíneo (BIRFS), em comparação com os exercícios de força de alta intensidade (AI) e de baixa intensidade sem restrição de fluxo sanguíneo (BI), sobre os níveis de subprodutos do óxido nítrico e sobre a atividade de enzimas antioxidantes em jovens saudáveis. Os principais resultados demonstram que: 1) uma sessão de exercício de força BIRFS não é capaz de modular os níveis plasmáticos de NOx; 2) o exercício de força BIRFS diminui significativamente a atividade da SOD e; 3) os exercícios de AI e BIRFS não modulam a atividade da CAT. 

 NOx são produtos finais do metabolismo do óxido nítrico, o qual exibe um papel fisiológico duplo, pois ao mesmo tempo em que desempenha função vasodilatadora, anti-hipertensiva e antiaterosclerótica, também pode causar danos oxidativos através da formação do radical peroxinitrito na presença do ânion superóxido 8 ^,^ 17 . Como anteriormente citado, os níveis plasmáticos de NOx não foram alterados em resposta à condição BIRFS. No entanto, quando comparados aos da condição AI, esses níveis se mostram significativamente maiores após o exercício. Já foi demonstrado que, agudamente, o exercício de força tradicional de alta intensidade pode comprometer a vasodilatação mediada pelo fluxo, reduzindo os níveis de NOx em indivíduos sedentários 18 ^,^ 19 . Nesse cenário, a redução da biodisponibilidade do óxido nítrico, associada à alta intensidade, pode ser resultado de dois fatores: aumento da ativação simpática durante o exercício e consequente vasoconstrição mediada pela acetilcolina 20 e aumento da preensão muscular sobre os vasos sanguíneos quando se recrutam grandes grupos musculares 19 ^,^ 20 . 

 Foi observada, no presente estudo, uma redução significativa da atividade da SOD na condição BIFRS. Como os níveis de NOx não se encontraram reduzidos após o protocolo BIRFS, pode-se sugerir que essa sessão não foi capaz de causar significativa produção de espécies reativas de oxigênio e consequente grau transitório de estresse oxidativo, não exigindo uma aumentada atividade da SOD para eliminação de um possível excesso de superóxido 8 ^,^ 10 . Nesse contexto, em um estudo envolvendo um protocolo de BIRFS muito semelhante, não foi encontrado aumento dos níveis sanguíneos de marcadores pró-oxidativos ou antioxidantes logo após a sessão de exercício, corroborando o fato de que esse tipo de sessão não é capaz de provocar significativo grau de estresse oxidativo 2 . 

 No presente estudo, a atividade da CAT não apresentou alteração frente aos protocolos BIRFS e AI. A enzima da CAT reduz os níveis de peróxido de hidrogênio (H_2_O_2_) à água (H_2_O) e ao oxigênio (O_2_) 10 . O baixo volume de exercício e a magnitude da lesão muscular podem justificar os resultados apresentados. No trabalho de Child et al., envolvendo um desenho experimental no qual os sujeitos realizaram 70 contrações excêntricas máximas de extensão de joelhos, foi encontrado aumento significativo de dano muscular, representado pela creatina quinase (CK) e, por consequência, aumento da proteção antioxidante 21 . Dessa forma, nossos achados também podem estar relacionados ao baixo impacto dos protocolos de exercícios sobre o dano muscular induzido pelo exercício. 

 Algumas limitações foram relevantes no presente estudo. Por exemplo, a medida da função vascular foi realizada apenas através de variáveis bioquímicas, sendo que medidas de análise de imagem ou pletismografia poderiam subsidiar inferências mais contundentes a partir de nossos resultados. Adicionalmente, a mensuração dos níveis plasmáticos de CK ou dos demais marcadores de lesão muscular poderiam dimensionar a magnitude metabólica das diferentes condições. 

## CONSIDERAÇÕES FINAIS

 A partir dos resultados apresentados, pode-se inferir que uma sessão de treinamento de força de baixa intensidade com restrição do fluxo sanguíneo não reduz a biodisponibiidade do óxido nítrico, bem como não induz desequilíbrio redox em indivíduos jovens saudáveis. Como perspectivas, futuros estudos devem avaliar de modo mais amplo a função vascular em resposta ao treinamento de força com restrição do fluxo sanguíneo.
